# Novel compound heterozygous *OBSCN* variants in Chinese children with congenital pulmonary airway malformation

**DOI:** 10.1186/s13052-025-01942-8

**Published:** 2025-03-28

**Authors:** Jiali Xu, Siyu Ma, Zhaocong Yang, Yang Xu, Jirong Qi, Min Da, Xuming Mo

**Affiliations:** Department of Cardiothoracic Surgery, Children’s Hospital of Nanjing Medical University, Jiangdong South No.8 Road, Nanjing, 210008 China

**Keywords:** CPAM, Compound heterozygous, *OBSCN*, Genetic mutations

## Abstract

**Background:**

Congenital pulmonary airway malformation (CPAM) belongs to rare lung abnormalities which may result in poor lung development outcomes. However, the etiology of CPAM has not been well described.

**Methods:**

In this study, whole-exome sequencing (WES) technology was used to sequence 9 CPAM families to screen the pathogenic genes and their mutations for CPAM. Sanger sequencing was performed for verify the functions of these mutations.

**Results:**

We found compound heterozygous mutations in *OBSCN* gene in two patients with CPAM: one with p.G8837A mutation inherited from her father and p.G1126S mutation inherited from her mother; the other with p.R5167W mutation inherited from his father and p.A7475T mutation inherited from his mother. Immunofluorescence results showed that the expression of OBSCN protein in the central lung tissue of CPAM patients was lower than that in the distal lung tissue of the lesion, and the expression of OBSCN protein was decreased in *OBSCN* mutated. We further explored the expression of *OBSCN* during mouse lung development, confirming that the gene mainly acts on the pseudoglandular stage.

**Conclusions:**

The *OBSCN* gene may play a key role in pseudoglandular stage during mouse lung development. The mutation of *OBSCN* gene may play a role in promoting the occurrence of CPAM, providing a possible therapeutic target for clinical intervention of CPAM.

## Introduction

Congenital pulmonary airway malformation (CPAM), which belongs to rare lung abnormalities, is characterized by excessive hyperplasia and dilation of terminal bronchioles during lung development [1]. A prospective study has shown that the incidence of CPAM is 1/27,400 between 1989 and 2008, but rose to 1/7,200 between 2009 and 2014 [2]. CPAM is caused by defective branching morphogenesis in the lung at different developmental stages and at different levels of the tracheobronchial tree, and is associated with respiratory distress in infants and potential malignancy in adults [3]. Familial clustering has been reported, suggesting a genetic predisposition to CPAM that may be caused by de novo or recessive mutations in genes that control airway development [4]. However, the genetic mechanism of CPAM has never been analyzed, due to sporadic CPAM cases ever reported. Therefore, to find CPAM pathogenic genes and mutations, we used whole-exome sequencing (WES) technology to sequence 9 CPAM families in the study.

## Materials and methods

### Study subjects

From October 2018 to January 2019, children with CPAM and their healthy parents were recruited who were admitted to the Department of Cardiothoracic Surgery, Children’s Hospital Affiliated to Nanjing Medical University, China. CPAM was diagnosed by surgery and pathology in all children. The CPAM classification criteria proposed by Stocker [5], was used in our study, which categorize these congenital lung lesions into five types (0–4) based on anatomical origin, cyst size, histology and clinical features: ① Type 0 (Tracheobronchial): Rare, involves acinar dysplasia, small cysts (< 0.5 cm) and poor prognosis; ② Type 1 (Bronchial): the incidence rate is 65–70%, large cysts (2–10 cm), bronchial epithelium, good outcomes after resected; ③ Type 2 (Bronchiolar): the incidence rate is 15–20%, small cysts (< 2 cm) and variable prognosis; ④ Type 3 (Bronchiolar/Alveolar Duct): the incidence rate is 5–8%, solid microcysts (< 0.2 cm), adenomatoid appearance; may cause respiratory distress in neonates; ⑤ Type 4 (Peripheral): the incidence rate is 5–10%, large thin-walled cysts, originates from distal acinus and overlaps with pleuropulmonary blastoma in some cases. Exclusion criteria: cases with other congenital lesions or known chromosomal aberrations; maternal gestational diabetes mellitus, eclampsia, maternal phenylketonuria, smoking during pregnancy, alcoholism, use of teratogenic drugs, history of exposure to chemical teratogens, etc. The study followed the tenets of the Helsinki Declaration and was approved by the Medical Ethics Committee of Nanjing Medical University. Informed written consent was obtained from the patient’s legal guardian.

### Whole-exome sequencing (WES) and sanger sequencing

Blood was sampled from the family pedigree and preserved in EDTA tubes. Genomic DNA was extracted from blood samples using the DNA Blood Min Kit (QIAGEN, Valencia, CA) following the manufacturer’s standard procedures. WES was conducted by Annoyouda Biotechnology Co. Ltd. Variant detection and genotyping were performed with GATK (https://software.broadinstitute.org/gatk/) and annotated with ANNOVAR. Common variants, such as intergenic, upstream, downstream, intronic, and synonymous variants, and variants with minor allele frequency (MAF) > 1% in the 1,000 genome, ExAC, and gnomAD databases, were filtered out. PolyPhen2, SIFT, Provean and phyloP were used to predict the impact of variants on protein function and structure. All potential variants were validated by Sanger sequencing. Primer-BLAST was used to design primers online. The primers were synthesized by Nanjing Qingke Biotechnology Co., Ltd., and listed in Table 1. Variants were amplified under an optimal condition for each primer pair, and then validated by Sanger sequencing using a machine of ABI-3500DX sequencer from Applied Biosystems Inc.

**Table 1:** Primers for Sanger verification of OBSCN mutations in patients with CPAM

Mutation	Primer	Sequence (5’-3’)
OBSCN c.3376 (exon11) G > A	chr1-1054-F	CAGGAACAGGGCAGGCTTGTG
	chr1-1054-R	AGAGGCAATTAGGTGGCACC
OBSCN c.15,499 (exon58) C > T	chr1-5973-F	GGATCTGTGCTTGTGAGCAC
	chr1-5973-R	GTATGTCTCCGTATCCAGCAG
OBSCN c.22,423 (exon97) G > A	chr1-4800-F	CTCCTTCTATGAGGTCAAGG
	chr1-4800-R	GCTCCGTGGAACAGAAGCCTC
OBSCN c.26,510 (exon115) G > C	chr1-6037-F	GACAGCCTTCATCATGTGAGTC
	chr1-6037-R	TCTGTTAGCCACGGGCACTG

CPAM: congenital pulmonary airway malformation

### Experimental animals

C57BL/6 mice (10 males and 20 females) were purchased from the Experimental Animal Center of Nanjing Medical University. After acclimatation to the environment, the mice were caged together (female to male ratio = 2:1). The day when the female mice presented vaginal plugs was set as the baseline date (0.5th day of gestational age [E 0.5]). We collected the lung tissues of mouse at the following eight time points: embryonic 10.5 days (E 10.5), embryonic 11.5 days (E 11.5), embryonic 13.5 days (E 13.5), embryonic 15.5 days (E 15.5), embryonic 18.5 days (E 18.5), postnatal 0.5 days (P 0.5), postnatal 3.5 days (P 3.5) and postnatal 11.5 days (P 11.5).

**Fig. 1:**
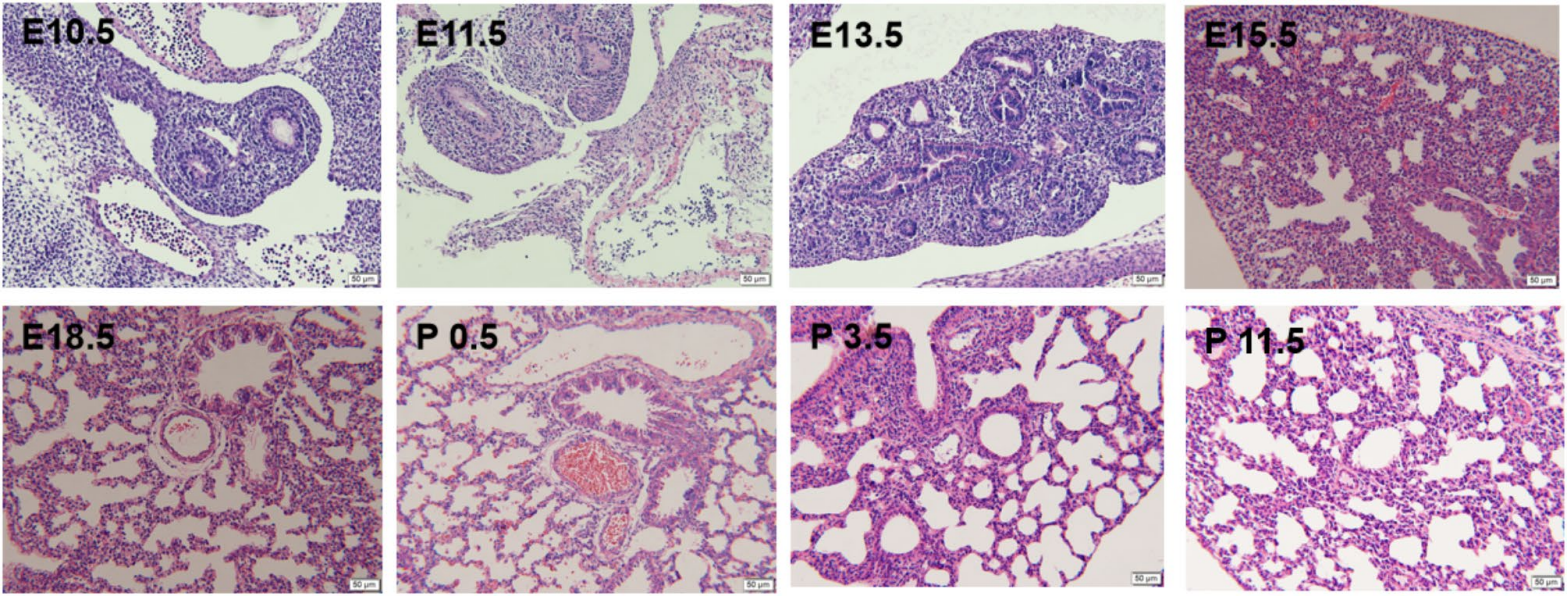
H&E staining of fetal and neonatal mouse lung tissues.

At E 10.5 and E 11.5 (embryonic stage), the ventral foregut of the endoderm proliferated to form two lung buds and continued to grow. At E 13.5 and E 15.5 (pseudoglandular stage), the bronchial trunk continued to branch into terminal bronchi, the vascular network built up, and the pulmonary arterial branches became parallel to the bronchial tree. At the canalicular stage, the lumens of the bronchi and terminal bronchioles were enlarged at E 18.5, P 0.5, and P 3.5, and the cuboidal epithelial cells differentiated into alveolar cells. At the saccular stage, the primitive alveoli were further divided and reconstructed, and the columnar epithelium gradually became a single cuboidal or squamous layer. The mesenchyme thinned, the capillaries abounded, and the capillary network formed up in the mesenchyme surrounding the alveoli rapidly. At P 11.5, the alveoli continued to differentiate and eventually matured (Fig. 1).

### RNA extraction and RT-PCR

Total RNA was extracted from lung samples of fetal or newborn mice using TRIzol reagent (Takara Biotechnology, Shiga, Japan). The qRT-PCR was performed using ChamQTM SYBR® qPCR Master Mix (Vazyme, Nanjing, China) on Roche LightCycler480. Primers for *OBSCN* were included: forward primer 5’-GCTCTGTGCTGGTCGTAGTG-3’ and reverse primer 5’-CCTCCTCGATGCCGTACTT-3’. The primers for *β-Actin* were forward primer 5’-GTGACGTTGACATCCGTAAAGA-3’ and reverse primer 5’-GCCGGACTCATCGTACTCC-3’.

## Results

A total of 9 CPAM families agreed to underwent WES, including 3 males and 6 females. Nine lesions (4 of type 1 CPAM and 5 of type 2 CPAM) included 1 in the right upper lobe, 2 in the left upper lobe, 5 in the right lower lobe, and 1 in the left lower lobe (Table 2).

**Table 2:** Clinical characteristics of patients with CPAM

Patient number	Gender	Age	Location	Pathological typing
1	Female	6 years	Right upper lobe	2
2	Female	7 months	Right lower lobe	1
3	Male	1 year	Right lower lobe	2
4	Female	13 years	Right lower lobe	1
5	Female	6 months	Left upper lobe	1
6	Male	1 year	Left upper lobe	2
7	Female	7 months	Right lower lobe	2
8	Female	8 years	Right lower lobe	2
9	Male	10 months	Left lower lobe	1

CPAM: congenital pulmonary airway malformation

CPAM is classified according to Stocker typing. Type I: It is composed of more than one cyst with a thick wall and a cyst diameter of 2–10 cm. The cyst wall is mainly composed of pseudostratified ciliated columnar epithelium. Type II: It is composed of multiple small cysts with a diameter of 0.5–2 cm and a wall of columnar or cuboidal epithelium

**Fig. 2:**
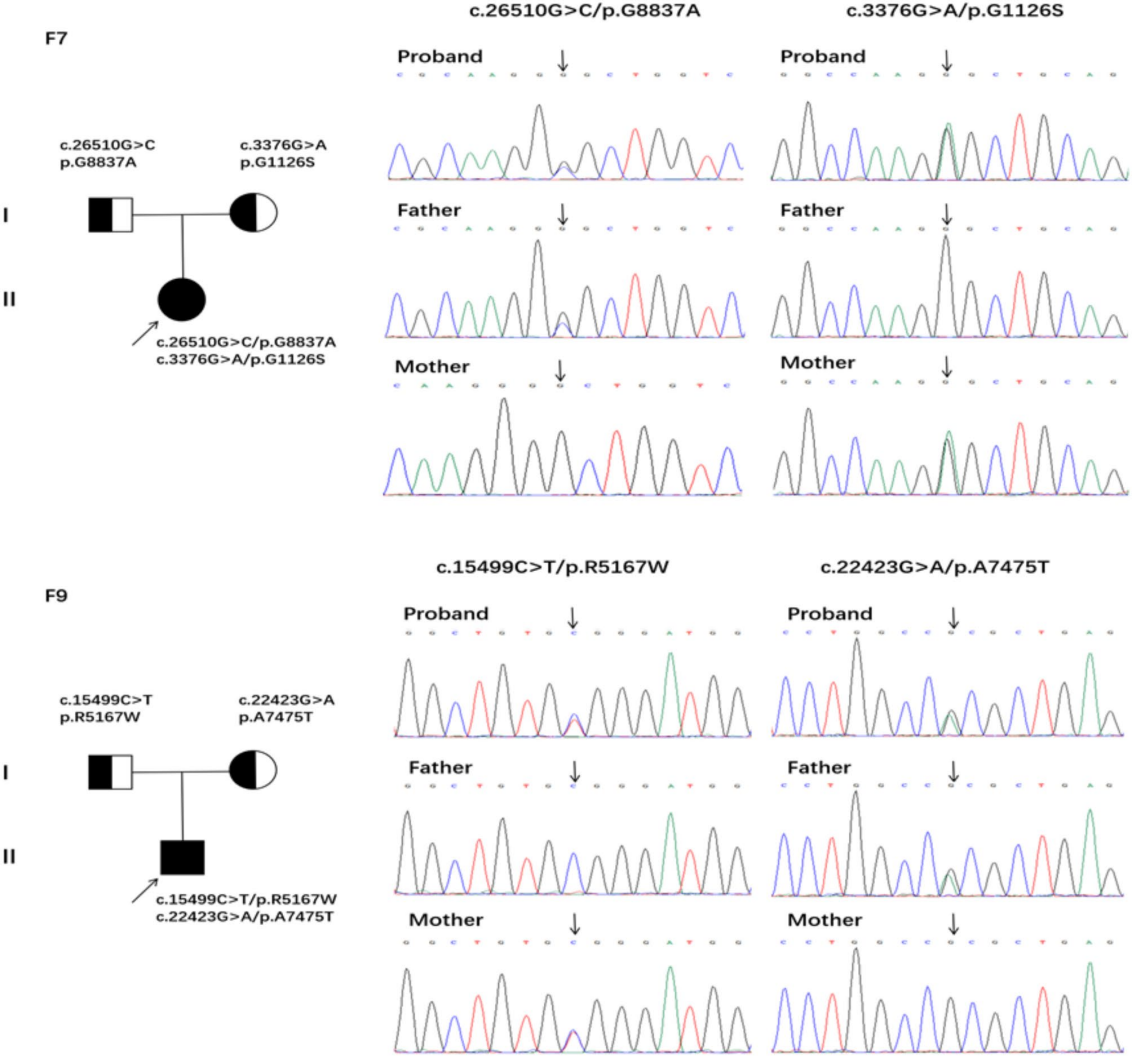
Compound heterozygous mutation in F7 family, OBSCN c.337(exon11)G>A from her mother, OBSCN c.26510(exon115)G>C from her father. Compound heterozygous mutation in F9 family, OBSCN c.15499(exon58)C>T from his mother, OBSCN c.22423(exon97)G>A from his father.

**Fig. 3:**
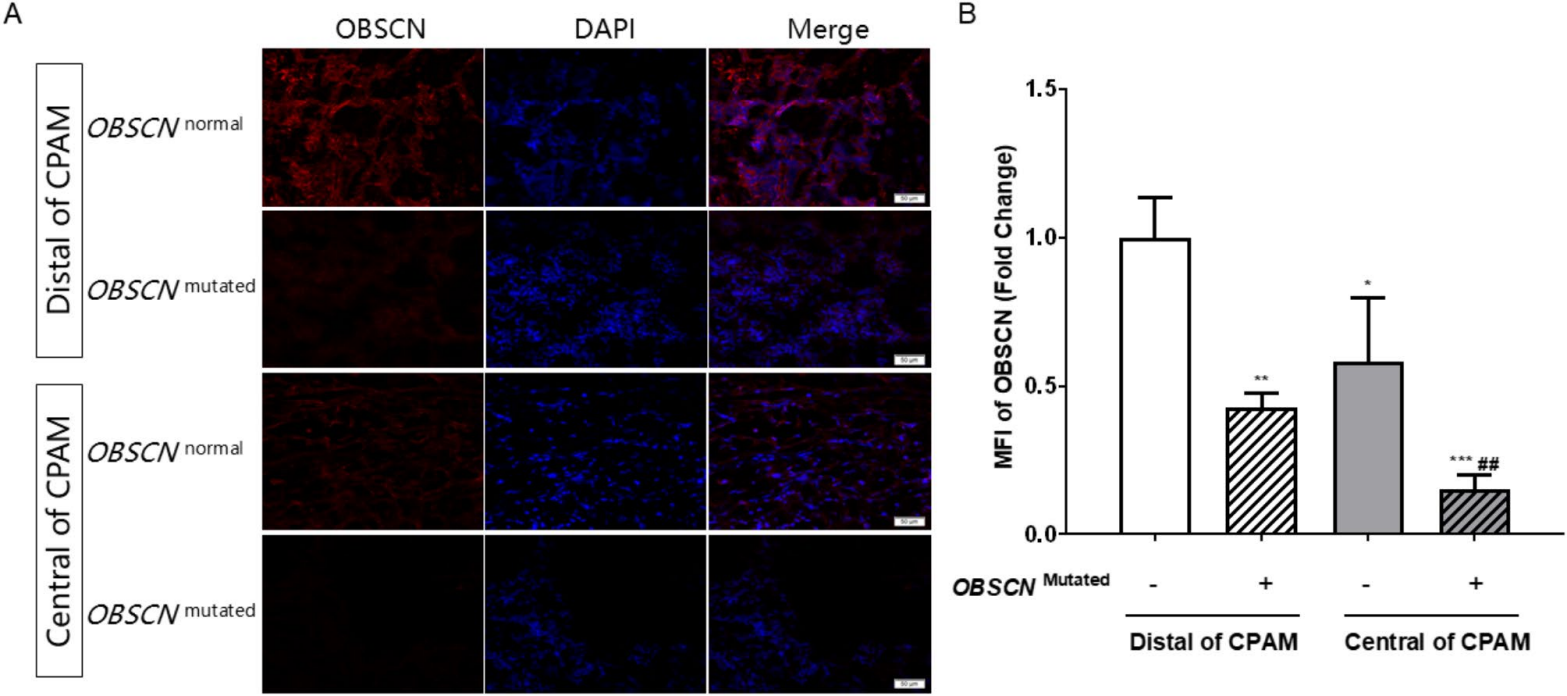
(**A**) Immunofluorescence staining of the central and distal of CPAM lesions of patients with and without *OBSCN* mutated. Red light is OBSCN and blue light is 4’,6-diamidino-2-phenylindole (DAPI). (**B**) Bar graph shows mean fluorescence intensity (MFI) of OBSCN staining. MFI of OBSCN fold change = MFI of OBSCN/ mean value of MFI of OBSCN in the control group (distal of CPAM without *OBSCN*^mutated^). ^*^p vs. distal of CPAM without *OBSCN*^mutated^; ^#^*p* vs. distal of CPAM with *OBSCN*^mutated^. ^*^, *p* < 0.05; ^**^, *p* < 0.01; ^***^, *p* < 0.001. ^##^, *p* < 0.01

We found compound heterozygous mutations in *OBSCN* gene in two cases: one with p.G8837A mutation from her father and p.G1126S mutation from her mother, the other with p.R5167W mutation from his father and p.A7475T mutation from his mother. All four mutations are rare in the Chinese population. The Sanger sequencing results were consistent with WES (Fig. 2). Four in silico programs, including PolyPhen2, SIFT, Provean and phyloP, revealed that four mutations could damage protein function and structure (Table 3). Immunofluorescence staining was performed on the central and distal of CPAM lesions of patients with and without *OBSCN* mutated, respectively. The results showed that the expression of OBSCN in the lung tissues with *OBSCN* mutated was decreased, and the expression of OBSCN in the central of CPAM lesions was lower than that in the distal lesion (Fig. 3).

**Table 3:** Functions of OBSCN mutations in patients with CPAM

Patient number	Variants	Effect	AF in ExAC	Allele Frequency in 1000 Genome Project	Polyphen2_HDIV	SIFT	Provean	phyloP
7	p.G1126S	nonsynonymous	0.0002		probably damaging (0.997)	damaging (0.029)	deleterious (-3.57)	deleterious (0.998000)
	p.G8837A	nonsynonymous	0		probably damaging (1.0)	tolerated (0.057)	deleterious (-2.82)	deleterious (0.977000)
9	p.R5167W	nonsynonymous	0.0011	0.0024	probably damaging (0.999)	damaging (0.019)	deleterious (-2.78)	deleterious (0.935000)
	p.A7475T	nonsynonymous	0.0004		benign (0.0)	tolerated (1.0)	neutral (1.25)	deleterious (0.183000)

CPAM: congenital pulmonary airway malformation

**Fig. 4:**
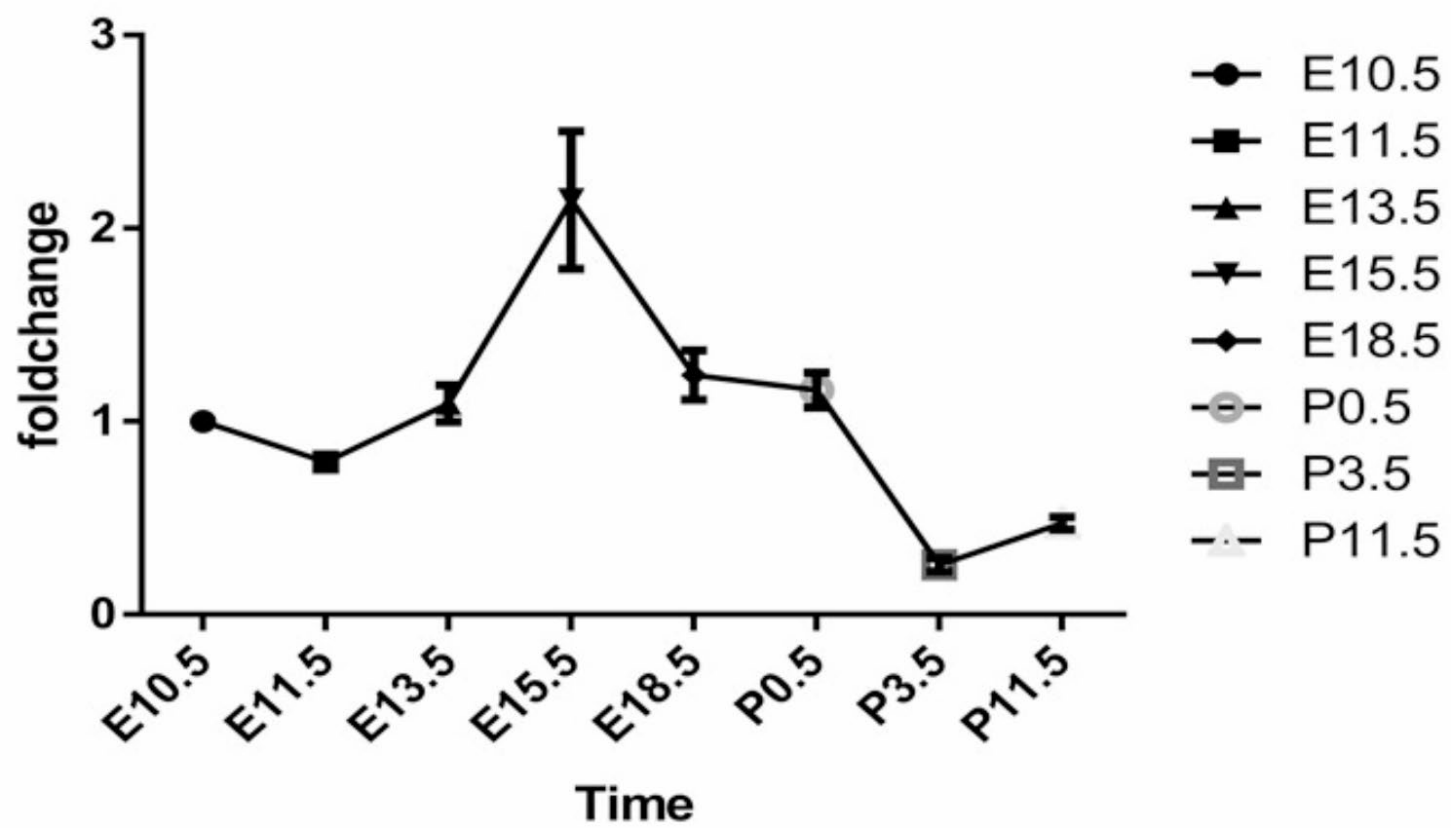
OBSCN expression in fetal and newborn mice lung tissues. qPCR showed that *OBSCN* expression increased rapidly in fetal lung tissue at E 13.5 and E 15.5

*OBSCN* mRNA expression was assessed in fetal and newborn mouse lung tissues. The absorbance of total RNA calculated as A260/A280 was 1.8-2.0. The expression level of *OBSCN* was normalized to the mRNA level of β-Actin from the same samples. *OBSCN* was expressed in all lung developmental stages of fetal and early postnatal mice. Among them, compared with that at E 10.5 and E 11.5, the *OBSCN* expression increased rapidly in fetal lung tissue at E 13.5 and E 15.5, and then gradually decreased to the trough at P 3.5 and P 11.5 after birth (Fig. 4).

## Discussion

In this study, we applied next generation sequencing (NGS) to find correlations between gene mutations and phenotypes of CPAM patients, with the expectation of providing adequate counseling for families (including more precise prognostic assessment and recurrence risk) [6–9], and further demonstrated that OBSCN gene plays a key role in lung development in animal experiments. We found compound heterozygous mutations of *OBSCN* gene in 2 families, including F7 c.3376(exon11) G > A; c.26,510 (exon115) G > C, F9 c.15,499 (exon58) C > T, c.22,423 (exon97) G > A. The *OBSCN* mutation results in decreased OBSCN protein expression of lung tissue in our study. The etiology of CPAM remains unveiled [10]. These mutations may play etiological roles in the development of CPAM in Chinese children. Compound heterozygous mutations can be inherited to cause familial diseases [11–13]. For the first time, our findings provide genetic clues into the pathogenesis of CPAM.

*OBSCN* gene (MIM# 84033) is located on human chromosome 1q42.13 and encodes a protein of approximately 720 kDa, originally discovered as a scaffold in striated muscle cells. OBSCN regulates the function of these cells during the development of diseases, such as hypertrophic cardiomyopathy (HCM), dilated cardiomyopathy (DCM) and left ventricular noncompaction (LVNC) [14–17]. Recent studies have shown giant OBSCN as a key player in cancer development and progression [18]. *OBSCN* mutates in the occurrence of solid tumors, such as breast cancer, colon cancer, glioblastoma, and melanoma [19–21], as well as their invasion and metastasis [22, 23].

Previous studies have shown that CPAM mainly develops at the pseudoglandular stage of lung development [24]. In our study, OBSCN expression was decreased in lung tissue at the center of the lesion in patients with CPAM compared with lung tissue distal to the lesion, and was further decreased in patients with *OBSCN* mutated. Therefore, in the present study, we performed H&E staining to analyze lung tissue sectioned at different lung developmental stages in wild-type fetal mice. At the same time, the expression of *OBSCN* gene at these stages was profiled. We found that *OBSCN* expression increased significantly in the fetal lung tissue at E 13.5, E 15.5 (pseudoglandular stage), compared to that at E 10.5 and E 11.5 (embryonic stage), then decreased from E 18.5 (cystic stage) at P 3.5 and P 11.5 (cystic stage to alveolar stage). Therefore, we speculated that OBSCN may play a key role at the pseutdoglandular stage of lung development. Thus, its mutation surely distorts the differentiation and formation of the bronchial tree, a mechanism implicated in the occurrence of CPAM.

Previous studies have shown the contribution of abnormal epithelial-mesenchymal transition (EMT) to the rise of congenital lung diseases [25, 26]. Meanwhile, OBSCN serves as a critical driver in EMT. Shriver et al. have shown that low-level OBSCN in mammary epithelial cells induces the EMT process, leading to disruption of cell-cell contacts and acquisition of a mesenchymal phenotype [27]. Therefore, OBSCN expression may be dysregulated to induce EMT, which ends up with CPAM. However, this mechanism should be verified by further research.

## Conclusions

The *OBSCN* gene may play a key role during mouse lung development, especially at its pseudoglandular stage. *OBSCN* mutated may play a role in promoting the occurrence of CPAM, providing a possible therapeutic target for clinical intervention of CPAM. However, the specific mechanism of *OBSCN* mutated in triggering CPAM needs to be further explored in molecular experiments.

## Data Availability

Data obtained in the present study are available upon request to the corresponding author.
